# Editorial: Innovations in industry 4.0: advancing mobility and manipulation in robotics

**DOI:** 10.3389/frobt.2026.1889141

**Published:** 2026-06-15

**Authors:** Kensuke Harada, Rajkumar Muthusamy

**Affiliations:** 1 Osaka University, Suita, Japan; 2 Dubai Future Foundation, Dubai, United Arab Emirates

**Keywords:** autonomous robotics, industrial automation, industry 4.0, intelligent grasping, intelligent navigation, mobile manipulation

## Introduction

Industry 4.0 represents a global shift towards interconnectivity, digitization, and automation to meet the evolving demands of modern industry. Robotics has become a fundamental component of Industry 4.0 technologies, finding applications across manufacturing, warehousing, retail, transportation, logistics, and various industrial processes. The integration of robotics aims to enhance operational efficiency, flexibility, and productivity. With ongoing digital transformation, increasing product variety and volume demands by consumers, and the adoption of smart manufacturing and logistics practices, there is a pressing need for autonomous robots in Industry 4.0 technologies. These robots must navigate complex environments, grasp and manipulate diverse objects in clutter, use effective end-tools, seamlessly integrate into automated workflows, learn and generalize skills, and collaborate safely with humans. In recent times, mobile manipulation has emerged as a crucial aspect, bridging static manipulation and navigation, and expanding the scope of achievable tasks in diverse settings. This Research Topic is dedicated to exploring and showcasing the latest innovations in robot mobility, manipulation, and mobile manipulation, specifically tailored to enable flexible and collaborative automation across diverse industrial sectors.

This Research Topic brings together nine contributions that collectively advance the state of the art across four major themes: intelligent grasping and manipulation, robot-robot collaboration and safety, robot navigation and localization, and quality inspection through deep learning. Together, they reflect the breadth and depth of current research efforts aimed at deploying capable, safe, and adaptive robotic systems in real-world industrial environments.

## Intelligent grasping and manipulation

### Collapse and collision aware grasping for cluttered shelf picking

Pathak et al. address a fundamental challenge in smart factory automation: safely retrieving objects from cluttered shelves without causing unintended collapses or collisions. Their system integrates dynamic physics simulation into the grasping pipeline, reconstructing an approximate 3D scene from a single RGB-D image to evaluate retrieval strategies before execution. Both heuristic-based and physics-based approaches are proposed for single-box extraction and full shelf clearance. Experiments on structured and unstructured box stacks demonstrate that the physics-aware method significantly outperforms baseline heuristics in success rate and efficiency.

### Visuo-tactile feedback policies for terminal assembly facilitated by reinforcement learning

Li et al. tackle the precision assembly of electrical terminal connectors, a task notorious for tight tolerances and damage-prone parts. They decompose the assembly into three phases—grasping, alignment, and insertion—each handled by a dedicated learning module. A vision-guided model picks the terminal head, a tactile-based pose estimation model aligns it with the base, and a visuo-tactile reinforcement learning policy executes the final insertion. Leveraging human demonstrations and safe RL training, the method achieves a 100% success rate across 100 test configurations, vastly outperforming imitation learning (9%) and online RL (0%) baselines.

### Vision-based manipulation of transparent plastic bags in industrial setups

Adetunji et al. address the challenging problem of autonomously cutting and unpacking transparent plastic bags, a common task in supply chain automation that is difficult due to the low visual contrast of transparent materials. Their system uses convolutional neural networks for robust bag detection under varying lighting conditions, combined with depth sensing and tracking algorithms for 3D spatial awareness. Compliance control with vacuum gripping handles the manipulation, and the entire system is validated on a FRANKA robot arm, demonstrating reliable performance for bulk-loader automation.

### Reliable and robust robotic handling of microplates via computer vision and touch feedback

Scamarcio et al. present a system for precise autonomous microplate handling in laboratory automation environments, where dynamic and variable conditions challenge conventional robotic systems. Their approach integrates SLAM, computer vision, and tactile feedback on a bi-manual mobile robot, achieving fine positioning accuracy of ±1.2 mm and ±0.4°. The framework generalizes well across different laboratory instruments and achieves at least a 95% success rate across varied conditions, highlighting its potential for enabling reliable, reproducible experimental workflows.

## Robot-robot collaboration and safety

### iAPF: an improved artificial potential field framework for asymmetric dual-arm manipulation with real-time inter-arm collision avoidance

Surya Prakash et al. introduce an improved Artificial Potential Field (iAPF) framework for controlling dual-arm robot manipulators in industrial component sorting. The system pairs the iAPF for linear velocity control with a Proportional-Derivative controller for angular velocity, creating a hybrid twist command that enables human-like asymmetric coordination. A priority-based state machine governs task allocation between arms, while a computationally efficient continuous distance calculation between robot links enables real-time collision monitoring. The integration of a YOLOv8-based vision system achieves 20 fps detection with 0.99/0.97 accuracy for bolt and nut detection, confirming practical viability.

### A multi-robot collaborative manipulation framework for dynamic and obstacle-dense environments

Adil et al. present a multi-robot collaborative manipulation framework implemented in Gazebo, designed for autonomous task execution in warehouse-like environments with dynamic obstacles and dense clutter. A leader-follower architecture governs coordination, and a centralized control structure based on MATLAB and Stateflow logic manages high-level scheduling. YOLOv2-based object detection on simulated RGB-D cameras identifies targets, while sampling-based path planning integrated with LiDAR data enables real-time obstacle avoidance and path rerouting. The framework scales with team size and demonstrates applicability to logistics automation and human-robot interaction scenarios.

## Robot navigation and localization

### Adaptive emergency response and dynamic crowd navigation for mobile robot using deep reinforcement learning

Alexander et al. address the problem of mobile robot navigation in high-density crowd and emergency scenarios using a Deep Reinforcement Learning framework. Three algorithms—DDPG, TD3, and DQN—are evaluated in a ROS2 Gazebo simulation environment using the TurtleBot3 platform. A context-aware state representation combining LiDAR-based obstacle perception, goal orientation, and robot kinematics enhances situational awareness. Extensive simulation results demonstrate that TD3 achieves the best performance across success rate, path efficiency, and collision avoidance, contributing a reproducible, constraint-aware DRL navigation architecture for real-time emergency applications.

### VIO-GO: optimizing event-based SLAM parameters for robust performance in high dynamic range scenarios

Sakhrieh et al. tackle a critical limitation in Industry 4.0 robotics: the poor performance of standard Visual Inertial Odometry (VIO) systems in dynamic and low-light industrial environments. Inspired by biological sensing, they integrate bio-inspired event cameras with conventional video and inertial data. A novel VIO-Gradient-based Optimization (VIO-GO) method employing Batch Gradient Descent (BGD) tunes Event SLAM parameters automatically using motion-compensated images. Experimental validation on the Event Camera Dataset demonstrates a 60% improvement in Mean Position Error over fixed-parameter methods, with the most comprehensive parameter set (VIO-GO8) reducing MPE by a further 24% compared to a minimal configuration.

## Deep learning for industrial inspection

### A systematic survey: role of deep learning-based image anomaly detection in industrial inspection contexts

Shukla et al. provide a comprehensive systematic review of deep learning methodologies for image anomaly detection in industrial manufacturing contexts. The survey evaluates traditional techniques against state-of-the-art supervised, unsupervised, and semi-supervised learning approaches, addressing challenges such as real-time processing constraints and imbalanced datasets. Popular anomaly detection datasets for surface defect detection are surveyed, and common evaluation metrics are critically examined. The review extends its scope to drone-based, manipulator-based, and AGV-based anomaly detection, offering valuable insights for researchers and practitioners developing intelligent quality inspection systems.

## Conclusion

The nine contributions assembled in this Research Topic collectively demonstrate the remarkable breadth of ongoing innovation at the intersection of robotics and Industry 4.0. From physics-aware grasp planning and multi-modal assembly policies to collaborative multi-robot frameworks and event-camera-based localization, these works push the boundaries of what autonomous robotic systems can achieve in complex, human-shared industrial environments. The integration of deep learning—whether for visual perception, reinforcement-based policy learning, or anomaly detection—emerges as a unifying thread across nearly all contributions, reflecting the central role that data-driven approaches now play in advancing robot intelligence.

Looking ahead, several open challenges remain. Safe and efficient human-robot interaction in unstructured environments, real-time adaptability to unexpected changes, and the generalization of learned skills across diverse tasks and platforms continue to demand creative solutions. The editors hope that this Research Topic will serve as both a record of current progress and a source of inspiration for the next-generation of research in mobile and manipulative robotics for Industry 4.0.

